# ECG Bootcamp - A Novel Emergency Medicine Small Group Session

**DOI:** 10.5070/M5.52155

**Published:** 2024-06-14

**Authors:** Kimberly Sokol, Anthony Lucero, Melissa Kemp, Nayana Singh, Ian Sprouse

**Affiliations:** * Sutter Roseville Medical Center, Department of Emergency Medicine, Roseville, CA; ^ Kaweah Health Medical Center, Department of Emergency Medicine, Visalia, CA; † University of Nevada, Reno School of Medicine, Reno, NV

## Abstract

**Audience:**

The target audience for this small group session is post-graduate year (PGY) 1–4 emergency medicine (EM) residents and medical students.

**Introduction:**

An electrocardiogram (ECG) is a crucial diagnostic tool in emergency medicine.^1^ Accurate ECG interpretation by emergency physicians is crucial for diagnosing life-threatening conditions, making it essential for EM residents’ training. The Accreditation Council for Graduate Medical Education (ACGME) lists ECG interpretation as a vital patient care milestone for residency graduation.^2^ Despite this, research suggests that few EM programs have standardized ECG curricula or test for ECG interpretation competency.^3^ To address this gap, we created the “ECG Bootcamp,” which uses gamification to engage learners in key ECG concepts through group learning.

**Educational Objectives:**

By the end of this group exercise, learners will be able to: 1) Recognize an emergent ECG that needs action immediately, 2) Demonstrate an understanding of normal vs abnormal pediatric ECGs, 3) Identify ECG manifestations of toxicologic conditions, 4) Describe the key characteristics of an ECG in a patient with a pacemaker and problems thereof, 5) Compare and contrast common ECG findings in patients with certain congenital/hereditary cardiac conditions, 6) Manage a patient presenting in extremis with torsades de pointes in the emergency department.

**Educational Methods:**

The “ECG Bootcamp” uses gamification, which incorporates game-design elements into educational activities to enhance learner engagement and motivation. This method was chosen to address the challenge of standardized ECG training in emergency medicine and the increasing use of gamification in graduate medical education.^4^–^6^ EM residency programs have implemented gamified curricula using quizzes, hands-on games, competitions, and longitudinal point-based games.^7^–^8^ By shifting from a passive lecture-based learning to a competitive format, the curriculum uses interactive learning to improve knowledge retention and participant satisfaction.^6^,^9^

Small group sessions were selected to ensure an intimate learning environment with a high faculty to student ratio. This setting allows for tailored instruction and immediate feedback, both of which are important for mastering ECG interpretation.

The bootcamp consists of six interactive stations, including competitive games and a simulation case. These stations are formulated to expose learners to important ECG concepts through different modalities, such as trivia and association games, while incorporating spaced learning to solidify understanding of cardiac pathology.

**Research Methods:**

The participants were provided with an anonymous online survey providing feedback regarding the quality of the educational content and the efficacy of the delivery method. The feedback had both a quantitative and qualitative component. The participants gave a rating between 1–10 out of 10, 1/10 being not effective learning, 10/10 being the most effective learning. In addition, they were given the option to leave comments.

**Results:**

A post-participation survey was provided to 46 participants, of which there were 18 respondents. We recognize that this is low and attribute this to the fact that the survey was sent out after the actual activity rather than included as part of the activity. One hundred percent of respondents rated both the small group game sessions and the simulation case a 10 out of 10 scale. Anonymous feedback comments from respondents were also provided.

**Discussion:**

The ECG Bootcamp serves as an effective model that addresses the gap in standardized ECG interpretation within emergency medicine residency programs. By integrating gamification with spaced repetition,^10^–^11^ this curriculum enhances learner engagement and aids in the solidification of cardiac pathology that often proves challenging in a traditional lecture format. Survey results indicated that the interactive, gamified nature of the session successfully meets the educational needs for learners across all levels of training.

Overall, this session provides a reproducible blueprint for residency programs aiming to improve their cardiovascular curriculum. Beyond ECG interpretation, the framework of rotating through gamified stations and simulations can be adapted to other clinical topics to stimulate a more dynamic and retentive learning environment in post-graduate medical education.

**Topics:**

Small groups, electrocardiogram (ECG), gamification, medical education.

## USER GUIDE

**Table:** 

List of Resources:	
Abstract	69
User Guide	71
Small Groups Learning Materials	74
Appendix A: Introduction to ECG Bootcamp	77
Appendix B: ECG Bootcamp Score Card	78
Appendix C: ECG Association Game PowerPoint	79
Appendix D: ECG Association Game Instructor Notes	80
Appendix E: Jeo-CARD-y Part 1 ECG Bootcamp PowerPoint	87
Appendix F: Jeo-CARD-y Part 1 ECG Bootcamp Instructor Notes	88
Appendix G: Jeo-CARD-y Part 1 ECG Bootcamp Score Sheet	93
Appendix H: Jeo-CARD-y Part 2 ECG Bootcamp PowerPoint	94
Appendix I: Jeo-CARD-y Part 2 ECG Bootcamp Instructor Notes	95
Appendix J: Jeo-CARD-y Part 2 ECG Bootcamp Score Sheet	101
Appendix K: Who Wants to be a Milliamp? ECG Bootcamp PowerPoint	102
Appendix L: Stimulus for Question 11 of Appendix K Presentation	103
Appendix M: Who Wants to be a Milliamp? ECG Bootcamp Instructor Notes	104
Appendix N: Code Blue Names Playing Cards	112
Appendix O: Code Blue Names Color Card	119
Appendix P: Code Blue Names Instructor Notes	120
Appendix Q: Code Blue Names Master Template	123
Appendix R: Torsades Simulation Case	124
Appendix S: Stimuli for Torsades Simulation Case	133
Appendix T: ECGs for Torsades Simulation Case	134
Appendix U: Answer Key for ECGs in Torsades Simulation Case	135
Appendix V: Torsades Simulation Case Score Sheet	137
Appendix W: ECG Bootcamp Curriculum Chart	139

**Learner Audience:**

This activity is appropriate for medical students, interns, and junior and senior residents.

**Time Required for Implementation:**

About 4 hours

**Recommended Number of Learners Per Instructor:**

4–6

**Topics:**

Small groups, electrocardiogram (ECG), gamification, medical education.

**Objectives:**

By the end of this group exercise, learners will be able to:

1. Recognize an emergent ECG that needs action immediately.
2. Demonstrate an understanding of normal vs abnormal pediatric ECGs.
3. Identify ECG manifestations of toxicologic conditions.
4. Describe key characteristics of pacemakers and associated problems.
5. Compare and contrast common ECG findings in patients with certain congenital/hereditary cardiac conditions.
6. Manage a patient presenting in extremis with torsades de pointes in the emergency department.

### Linked objectives and methods

The “ECG Bootcamp” achieves its goals by integrating clinical objectives into a gamified, small group framework. This method was selected to transition from passive, lecture-based learning to an active participation model. With a high faculty to student ratio, the session ensures an intimate environment for learning.

The curriculum maps the six educational objectives to the following stations:

Objective 1: Recognize an emergent ECG that needs action immediately.
  Stations: ECG Association Game, ECG Jeo-Card-y Part 1 and ECG Jeo-Card-y Part 2. These stations focus on rapid recognition of critical patterns, such as STEMIs and De Winter’s T waves, and require learners to associate these findings with clinical interventions.
Objective 2: Demonstrate an understanding of normal vs abnormal pediatric ECGs.
  Stations: Who Wants to Be a Milliamp and ECG Jeo-Card-y Part 2. Using a trivia format, these stations cover pediatric-specific nuances like axis deviations and age-appropriate heart rates.
Objective 3: Identify ECG manifestations of toxicologic conditions.
  Station: ECG Jeo-Card-y Part 2 and Code Blue Names. Learners will be challenged to identify important toxic syndromes like digoxin effects from a patient’s ECG.
Objective 4: Describe key characteristics of pacemakers and associated problems.
  Stations: ECG Jeo-Card-y Part 1 and Who Wants to Be a Milliamp. These games utilize progressive difficulty to teach pacemaker characteristics including capture vs sensing issues.
Objective 5: Compare and contrast common ECG findings in congenital/hereditary cardiac conditions. Stations: Code Blue Names and ECG Jeo-Card-y Part 1. These stations will help learners solidify diagnostic criteria for conditions like Brugada syndrome.
Objective 6: Manage a patient presenting in extremis with torsade de pointes in the ED.
  Station: Simulation Case. This station requires integration of all previous objectives into a clinical scenario, helping learners to recognize an unstable rhythm and execute immediate clinical interventions.

### Recommended pre-reading for facilitator

Faculty moderators are expected to read the instructions for their assigned station and familiarize themselves with the educational content that is included. The instructions and educational content are included in this manuscript.

### Learner responsible content (LRC)

Learners are expected to have a baseline proficiency in ECG interpretation including rhythm identification, axis, and intervals.

### Small group application exercise (sGAE)

See the following attached materials for this small group exercise

- Appendix A: Introduction to ECG Bootcamp
- Appendix B: ECG Bootcamp Score Card
- Appendix C: ECG Association Game PowerPoint
- Appendix D: ECG Association Game Instructor Notes
- Appendix E: Jeo-CARD-y Part 1 ECG Bootcamp PowerPoint
- Appendix F: Jeo-CARD-y Part 1 ECG Bootcamp Instructor Notes
- Appendix G: Jeo-CARD-y Part 1 ECG Bootcamp Score Sheet
- Appendix H: Jeo-CARD-y Part 2 ECG Bootcamp PowerPoint
- Appendix I: Jeo-CARD-y Part 2 ECG Bootcamp Instructor Notes
- Appendix J: Jeo-CARD-y Part 2 ECG Bootcamp Score Sheet
- Appendix K: Who Wants to be a Milliamp? ECG Bootcamp PowerPoint
- Appendix L: Stimulus for Question 11 of Appendix K Presentation
- Appendix M: Who Wants to be a Milliamp? ECG Bootcamp Instructor Notes
- Appendix N: Code Blue Names Playing Cards
- Appendix O: Code Blue Names Color Card
- Appendix P: Code Blue Names Instructor Notes
- Appendix Q: Code Blue Names Master Template
- Appendix R: Torsades Simulation Case
- Appendix S: Stimuli for Torsades Simulation Case
- Appendix T: ECGs for Torsades Simulation Case
- Appendix U: Answer Key for ECGs in Torsades Simulation Case
- Appendix V: Torsades Simulation Case Score Sheet
- Appendix W: ECG Bootcamp Curriculum Chart

### Results and Tips for Successful Implementation

The quantitative success of ECG Bootcamp demonstrated strong learner acceptance of gamification as an educational strategy for improving ECG interpretation skills and reinforcing competency within the ACGME milestone framework. All survey respondents rated both the educational sessions and simulation components a 10 out of 10, suggesting a high degree of learner engagement, perceived educational value, and satisfaction with the curriculum structure. These findings supported the use of interactive, game-based learning as an effective supplement to traditional ECG instruction within emergency medicine residency education.

A total of 18 of the 46 participants completed the postcurriculum survey. We attributed the lower response rate to the survey being distributed after the completion of the bootcamp day rather than during the educational session itself. Based on this experience, we recommend that future implementations of similar curricula incorporate protected time immediately following the conclusion of the bootcamp for learners to complete evaluations prior to participant dismissal and award distribution. This approach may improve survey completion rates and allow for more comprehensive feedback collection.

Given the overwhelmingly positive quantitative results, qualitative feedback became the primary mechanism for identifying opportunities for curricular refinement and iterative improvement. Although overall feedback was highly favorable, the “Code Blue Names” station was consistently identified as the least preferred activity. Participants reported that the complexity of the game rules occasionally distracted from the educational objectives and created unnecessary cognitive load during gameplay. In response to this feedback, the curriculum was modified to include a standardized instruction guide and a brief orientation video for both educators and learners to review prior to gameplay. These additions were intended to improve learner understanding of the rules, decrease time spent clarifying gameplay mechanics, and maximize time devoted to ECG interpretation and discussion.

Qualitative analysis of the simulation station also revealed a strong learner preference for incorporating hands-on interaction with the simulation equipment, particularly the opportunity to perform chest compressions and participate in active resuscitation scenarios. Participants noted that these components increased realism and enhanced the connection between ECG recognition and clinical decision-making during cardiac emergencies. Based on this feedback, subsequent iterations of the curriculum emphasized the use of fully functional simulation equipment and integrated higher-fidelity clinical scenarios to better bridge the gap between theoretical ECG knowledge and practical bedside application.

Learners additionally reported that rotating through six different instructors was a major strength of the curriculum. Participants valued exposure to multiple teaching styles, approaches to ECG interpretation, and varied clinical perspectives. Several respondents specifically noted that hearing similar concepts reinforced in different ways across stations improved retention and strengthened understanding of difficult ECG patterns. Learners also highlighted that the repeated exposure to core concepts across multiple stations created a form of spaced reinforcement that enhanced long-term recall. In response to this feedback, the timing of ECG Bootcamp within the residency curriculum was intentionally adjusted so that the experience now serves as a structured refresher immediately preceding PGY-3 competency examinations, further leveraging spaced repetition principles to reinforce ECG interpretation skills prior to formal assessment.

## Supplementary Information



























## Figures and Tables

**Figure:**
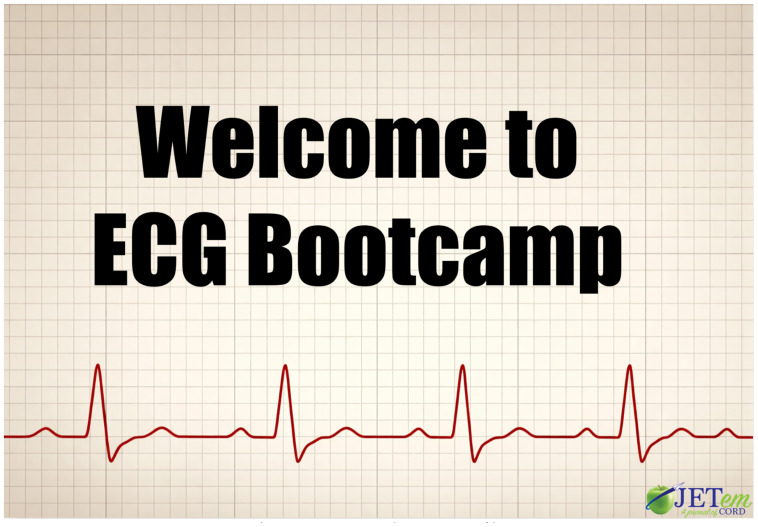
Please see associated PowerPoint file

**Figure:**
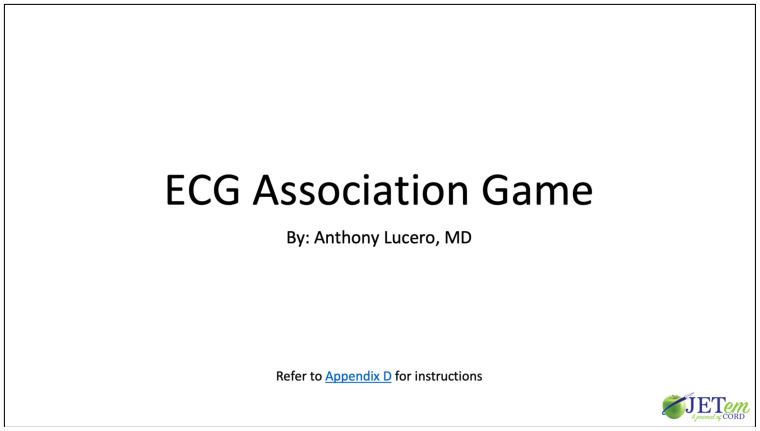
Please see associated PowerPoint file

**Figure:**
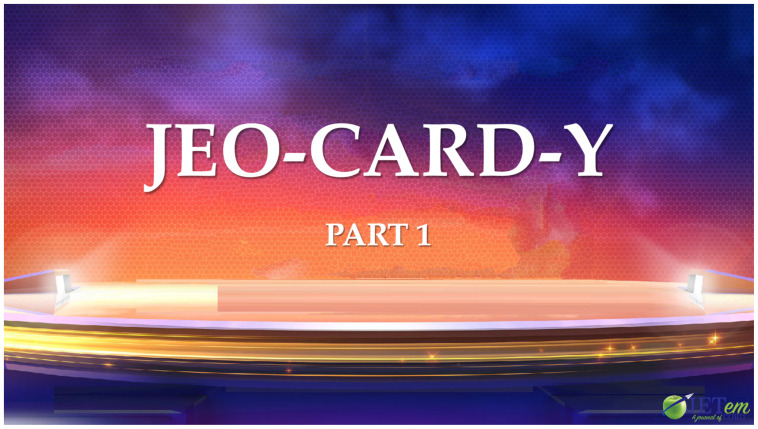
Please see associated PowerPoint file

**Figure:**
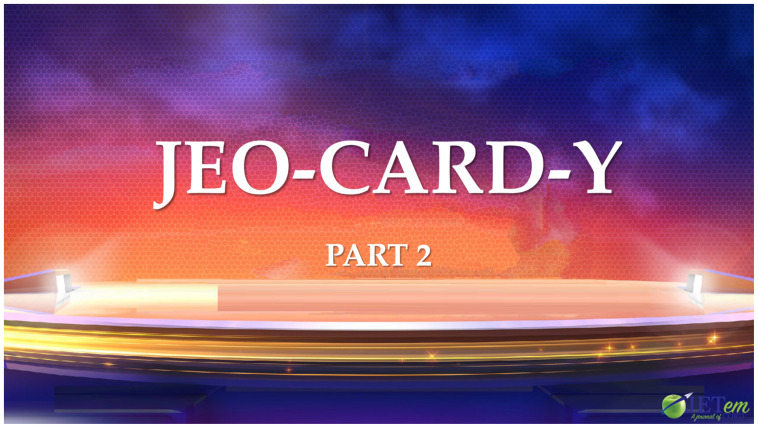
Please see associated PowerPoint file

**Figure:**
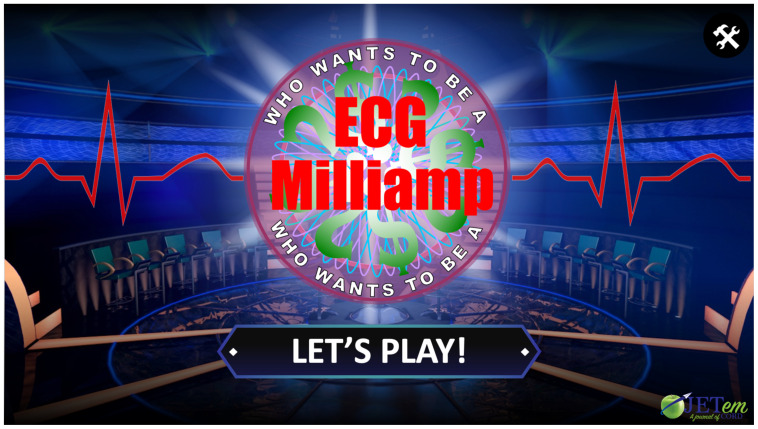
Please see associated PowerPoint file

**Figure:**
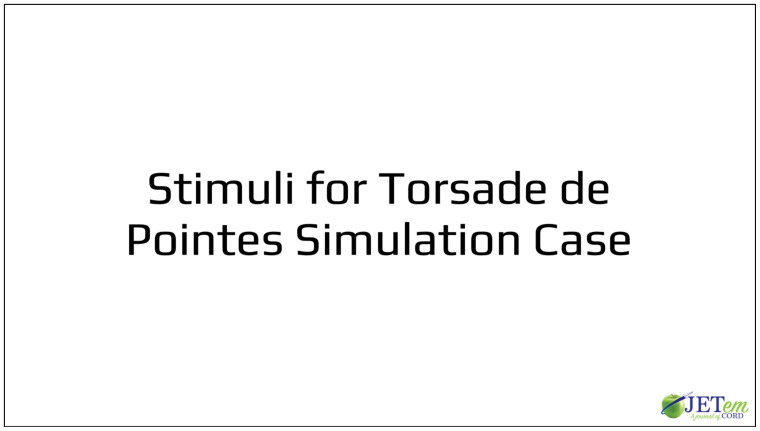
Please see associated PowerPoint file

**Figure:**
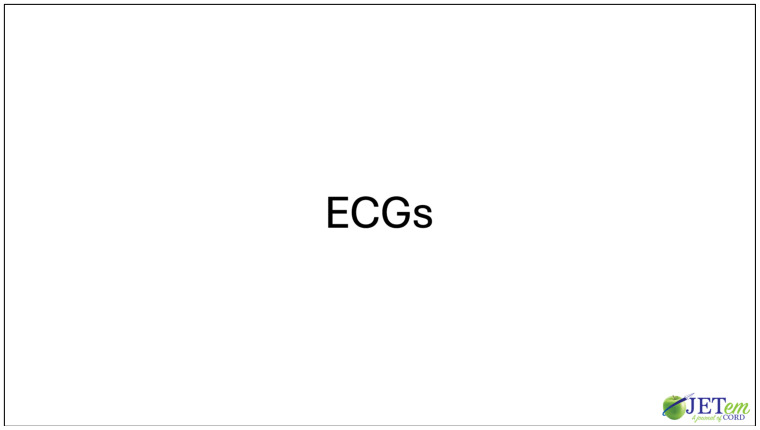
Please see associated PowerPoint file

## SMALL GROUPS LEARNING MATERIALS

### Small Group Application Exercise (sGAE)

**Small group application exercise (sGAE):**

The bootcamp is conducted over approximately four hours and begins with a ten-minute introduction designed to orient participants to the expectations and structure of the small group sessions. During this introduction, the lead facilitator outlines the schedule, explains the gamification elements, and divides learners into six teams. Group sizes are intentionally kept small to maximize engagement and participation, with a minimum of two learners and a maximum of eight learners assigned to each station. This introduction ensures all participants understand the objectives of the bootcamp and the thirty-five-minute rotation format.

Prior to the start of the bootcamp, faculty moderators are assigned to specific rooms and are responsible for ensuring that all educational materials, including presentations and game materials, are prepared before the start of the session. Each station follows a standardized workflow consisting of approximately three minutes dedicated to explaining the game mechanics, followed by thirty-two minutes of active gameplay and facilitated learning activities. These materials are utilized across six rotations:

1. ECG Association Game (Appendices C and D): Learners must associate ECGs laid out on a four-by-four grid that corresponds to a clinical clue provided by the educator. This requires rapid pattern recognition of ECG interpretation.
2. ECG Jeo-Card-y Part 1 (Appendices E, F and G): This station uses a trivia format where ECGs are presented as clues. Learners must identify the correct pattern, diagnosis, or mimic. Categories include “Indiana Pacemakers” (pacer complications), “STEMI Mimics,” and “Drugs and Bugs” (drug- and infection-induced ECG changes).
3. ECG Jeo-Card-y Part 2 (Appendices H, I and J): In this second trivia station, ECG clues and clinical scenarios are paired. Learners must identify the correct rhythm or pathology within categories of “Shock, Block, or Neither,” “The Real Deal” (True STEMIs), and “Nature vs Nurture” (distinguishing normal variants from actual pathology).
4. Who Wants to Be a Milliamp? (Appendices K, L and M): This is an interactive quiz session where learners answer fifteen progressively challenging ECG interpretation questions. Concepts range from basic rhythm identification to complex arrhythmias, utilizing “lifelines” to encourage collaboration.
5. ECG Code Blue Names (Appendices N, O, P and Q): Teams work to match clinical clues to the correct ECG pathology cards.
6. Simulation Case (Appendices R, S, T, U and V): Learners manage a simulation patient presenting in extremis with torsades in the emergency department.

The full stimulus sets for each station are provided in the supplemental files.

**Stations:**

The following instructions outline the requirements of each of the six rotating modules. All associated presentations, documents, and detailed instructions are provided as separate supplemental files.

1) ECG Association Game (Appendices C and D)
  Materials: 4 x 4 grid of sixteen printed ECG cards, Instructor clue sheet, Instructor answer key.
  Setup: Lay each of the 16 cards face up in a grid on a central table.
  Instructions: The instructor reads a clinical clue, and the team must identify the corresponding ECG as quickly as possible.
2 & 3) ECG Jeo-Cardy Part 1 & 2 (Appendices E–J)
  Materials: Laptop with PowerPoint, Room with projector, Instructor answer key.
  Setup: Ensure the PowerPoint is in presenter mode so the score board is visible.
  Instructions: Teams select categories and point values and provide their response to the stimuli as a question.
4) Who Wants to Be a Milliamp? (Appendices K–M)
  Materials: Laptop with PowerPoint, Room with projector, Instructor answer key.
  Setup: Display the PowerPoint in presenter mode.
  Instructions: Teams work through 15 progressively difficult questions. If they miss a diagnosis without a lifeline, they lock in their score at the current point level.
5) ECG Code Blue Names (Appendices N–Q)
  Materials: 25 printed matching cards, 5 × 5 master game board, Instructor Code Blue Master Template, Instructor answer key.
  Setup: Lay out the 25 cards in a 5 × 5 grid.
  Instructions: Divide the teams into clue givers and guessers. Team members will give clues to the guessers in order to guess the correct pathology card.
6) Simulation Case (Appendices R–V)
  Materials: Manikin with cardiac monitor, mock medications, simulation case, stimuli for simulation case.
  Setup: See supplemental files for specific simulation center set-up.
  Instructors: Recommend two: one to help run the case, and the other to be an embedded actor providing participants with ECGs of other “patients” in the department for their interpretation.

**sGAE Answers:**

Detailed answer keys and facilitator guides for each of the six rotations are provided as supplemental files. These documents ensure standardized scoring and allow faculty moderators to explain consistent clinical findings across different sessions.

**Brief wrap up (optional):**

Following the final rotation, a ten-minute large group session is held to conclude the bootcamp. The lead facilitator announces the final team scores and awards the winning team. This time is used to address any remaining questions before the ECG Bootcamp concludes.

## Appendix D ECG Association Game Instructor Notes

General notes to the Educator:

1. The object of the game is to “associate” ECGs together based on a prompt you give the learners. Depending on the prompt, there will be anywhere between 2–4 ECGs that can be associated with one another.
  - The prompt you give the group will be the combination of two things:
    - A word or phrase.
    - A number indicating how many ECGs apply to that word or phrase. Learners must select exactly that number of ECGs and may not exceed it.
      1. Example: You say: “Environmental/Toxicology, 3,” and the learners should choose Bi-directional Vtach (Dig toxicity), Slow regularized AF (Dig toxicity), and Osborne J waves (hypothermia).
  - This game is similar in structure to the game *Code Names*R, except there aren’t two teams playing against each other. It will simply be you, the Educator, giving them pre-determined prompts, and the learners collaborating to determine which ECGs can be “associated” together to fit the prompt.
2. Educational Goals:
  - Correctly identify each of the 16 ECGs.
  - Be able to associate them via electrocardiographic patterns or underlying pathophysiology.
3. Timing:
  - You have 35 minutes to go through 10 associations with an optional bonus round at the end, so please keep this at about 2–3 minutes per association. If the learners cannot get it in that amount of time, you should call time and record what was chosen.

Game Preparation:

1. Print out all 16 ECGs located on ECG Association Game PowerPoint.
2. Lay them out in 4×4 grid.

Game Instructions:

1. Read out associations with number of corresponding ECGs seen on “ECG Association Game Key” below.
2. The entire group will collaboratively point to or verbally select ECGs from the 4×4 grid; ECGs may be reused across rounds.
3. Score the group out of 3 based on how many ECGs are correctly identified (All are correct = 3 points, all but one correct = 2 points, at least one correct = 1 point, none correct = 0 points.)
  - Example 1: You say, “Ischemia/Infarction, 3,” and they correctly choose Anterolateral STEMI and aVR elevation MI but incorrectly choose hyperkalemia. Then the group gets 2 points.
  - Example 2: You say, “Wide complex tachycardia, 4,” and they correctly choose Monomorphic Vtach and SVT with aberrancy but incorrectly choose Brugada and WPW. Then the group gets 1 point.
4. Provide teaching points (located in the note section of corresponding slides). Each ECG has an explanation that you can teach the learners about that ECG.
5. Repeat until all 10 associations are completed.
6. Complete the 11th association as a bonus round (no score given).
7. Calculate and record the final score at the end of each session on each team’s scorecard, so that a final winner for ECG boot camp can be determined!

ECG Association Game KEY:

Association #1: “Peaked T, **2**”

1. De Winter’s T waves (slide 2)
2. Hyperkalemia (slide 3)

Association #2: “Wide complex tachycardia, **4**”

1. Monomorphic Vtach (slide 4)
2. SVT with aberrancy (RBBB) (slide 5)
3. Bi-Ventricular Vtach (slide 6)
4. Torsade de Pointes (slide 11)

Association #3: “Ischemia/Infarction, **3**”

1. De Winter’s T waves (slide 2)
2. Anterolateral STEMI (slide 13)
3. aVR elevation MI (slide 14)

Association #4: “Inherited structural/electrical abnormality, **3**”

1. Brugada (type I) (slide 10)
2. Hypertrophic cardiomyopathy (slide 15)
3. WPW (slide 16)

Association #5: “Family Hx of sudden cardiac death at early age, **3**”

1. Torsade de Pointes (slide 11)
2. Hypertrophic cardiomyopathy (slide 15)
3. WPW (slide 16)

Association #6: “Atrial abnormality, **2**”

1. Slow, regularized AF (slide 7)
2. Atrial flutter with variable block (slide 17)

Association #7: “Environmental/Toxicologic, **3**”

1. Bi-Ventricular Vtach (slide 6)
2. Slow, regularized AF (slide 7)
3. Osborn J waves (slide 12)

Association #8: “AV node abnormality, **3**”

1. SVT with aberrancy (RBBB) (slide 5)
2. Second degree type I AV block (slide 8)
3. First degree AV block (slide 9)

Association #9: “Ventricular abnormality, **4**”

1. Monomorphic Vtach (slide 4)
2. Bi-Ventricular Vtach (slide 6)
3. Torsade de Pointes (slide 11)
4. Hypertrophic cardiomyopathy (slide 15)

Association #10: “STEMI mimic, **3**”

1. Hyperkalemia (slide 3)
2. Brugada (type I) (slide 10)
3. Osborn J waves (slide 12)

Association #11: “STEMI equivalent, **2**”

1. De Winter’s T waves (slide 2)
2. aVR elevation MI (slide 14)

*** Note that Anterolateral STEMI on slide 13 doesn’t count since this is a classic STEMI

Presentation notes:

Slide 2 - De Winter’s T wave

- Upsloping ST depression in the precordial leads (> 1mm at J point)
- Peaked anterior T waves (V2–6), with the ascending limb of the T wave commencing below the isoelectric baseline
- Subtle ST elevation in aVR > 0.5mm

Slide 3 - Hyperkalemia

- Tall, symmetrically peaked T waves
- No preceding ST depression like in DeWinter’s

Slide 4 - Vtach

- Classic monomorphic VT with uniform QRS complexes
- Indeterminate axis
- Very broad QRS (~200 ms)

Side 5 - SVT with aberrancy

- Underlying right bundle branch morphology (RsR’)

Slide 6 - Bi-Ventricular V Tach (Digoxin toxicity)

- Alternating QRS axis with each beat

Slide 7 - Slow regularized Atrial Fibrillation (Digoxin toxicity)

- Coarse AF (irregular baseline)
- Regular narrow complex rhythm at 60 bpm
- The combination of atrial fibrillation with a regular rhythm (“regularized AF”) indicates that none of the atrial impulses are conducted to the ventricles, i.e. complete heart block is present
- The narrow complex rhythm is therefore a junctional escape rhythm

Slide 8 - Mobitz Type I, 2^nd^ Degree Heart Block

- Progressive prolongation of PR interval, with a subsequent non-conducted P wave
- Repeating 5:4 conduction ratio of P waves to QRS complexes
- Relatively constant P-P interval despite irregularity of QRS complexes

Slide 9 - 1^st^ Degree Heart Block

- PR interval is 300 ms
- Normal interval 200 ms

Slide 10 - Brugada (Type 1)

- Coved ST segment elevation >2 mm in >1 of V1–V3 followed by a negative T wave

Slide 11 - Torsade de Pointes

- “Twisting of Peaks”
- A type of polymorphic Vtach with progressively increasing then decreasing amplitude
- Classically associated with prolonged QT

Slide 12 - Osborn J waves

- Bradycardia
- Notching at the J point (seen in V2–6)
- Long QT interval (~ 600 ms)
- Shivering artifact

Slide 13 - Anterolateral STEMI

- ST elevation in V2–6, I and aVL
- Reciprocal ST depression in III and AVF

Slide 14 - aVR elevation MI

- ST horizontal/down sloping ST depression in multiple leads (V3–6, I, II, aVL)
- ST elevation in aVR > V1
- Recall: ST elevation must be > 1 mm with reciprocal depressions, AND have a history indicating ACS to meet criteria

Slide 15 - Hypertrophic Cardiomyopathy

- Voltage criteria for LVH in precordial and limb leads (LVH criteria: *Sokolov-Lyon criteria*: S wave in V1 or V2 + tallest R wave in V5–V6 > 35 mm)
- Narrow, “dagger-like” Q waves in inferior and lateral leads

Slide 16 - WPW

- Short PR interval (< 120 ms)
- Delta wave - Broad QRS complexes with a slurred upstroke to the QRS complex
- Negative delta wave in aVL simulating the Q waves of an old MI—referred to as the “pseudo-infarction” pattern

Slide 17 - Atrial flutter with variable block

- Inverted flutter waves in II, III + aVF with atrial rate ~ 300 bpm
- The degree of AV block varies from 2:1 to 4:1

## Appendix F Jeo-CARD-y Part 1 ECG Bootcamp Instructor Notes

**Notes to the Educator (PLEASE READ):**

*****This slideshow was created in PowerPoint and works best with PowerPoint features. It is fully viewable in Google Slides, but some animations, transitions, or formatting may not display exactly as intended. Please download it before opening it in Google slides.** Please download it to PowerPoint before opening it in Google slides.*******

1. Only half of the board is shown. This gives you 35 minutes to go through 15 prompts and allows time for teaching after each one. There is another station where the other 3 categories of Jeo-card-y are played.
2. Before you start, please divide each group into 2–3 separate smaller groups.
  - The reason for this is to encourage collaboration between group members.
  - Please make sure that each group has an even distribution of medical students, EM PGY-1s, 2s, 3s, and TY/Anesthesia PGY-1s.
3. Each of these 3 smaller groups will be a “contestant.” To make this more fun, each “contestant” can choose their own joke name and write it on a sheet of paper.
4. Randomly select which “contestant” goes first. They will choose a square on the board.
5. You, the educator, must be allowed to read the prompt in its entirety **BEFORE** a contestant is allowed to answer. If a contestant attempts to answer before you finish the prompt, they will be ignored.
6. For a contestant to indicate they are ready to answer the prompt, they must tap the desk in front of them. Whichever contestant you recognize first is allowed to answer.
7. The answer to the prompt **must be in the form of a question**. Those answers that are not in the form of a question will be deemed incorrect.
8. Each correct answer adds the $ amount associated with the prompt to the contestant’s score. Each incorrect answer subtracts the $ amount associated with the prompt from the contestant’s score.
9. After each prompt is finished, please click “click to return to Jeo-card-y board” or the home button at the bottom to return to the game board. If you click anywhere else on the screen, it will advance you to the next prompt without allowing the contestant to choose which they want.
10. At the end of the game, the scores should be calculated as shown:
  **Table:**
  Raw Score	Points (out of 30)
  0–500	10
  600–1000	15
  1100–1500	20
  1600–2000	25
  2000+	30
11. At the end of each session, the mini team with the highest score will receive candy, and their score will be recorded for their larger team (4–6 players). These scores should be recorded and eventually summed across all stations to determine the final winning team for ECG Bootcamp.

**Answer Key:**

**It’s a trap! (STEMI mimics)**

- **$100 - Answer: What is hyperkalemia?**
  ○ Peaked (tall, narrow) T waves - often “tented” in appearance
- **$200 - Answer: What is LVH with strain?**
  ○ Markedly increased QRS voltages - the S waves in V3 are so deep they are literally falling off the page!
  ○ ST elevation in V1–3 is in proportion to the very deep S waves (“appropriate discordance”)
  ○ LV strain pattern is seen in all leads with a positive R wave (V5–6, I, II, III, aVF)
- **$300 - Answer: What is Type 1 Brugada?**
  ○ Will also accept: What is Brugada?
  ○ Sodium channelopathy
  ○ ECG changes can be “unmasked” or augmented by a number of stressors:
    ■ Drugs/ETOH
    ■ Fever
    ■ Hypokalemia
    ■ Hypothermia
    ■ Many anti-arrhythmic meds (sodium CCB [calcium channel blocker], calcium CB [channel blocker], beta blocker, nitrates)
- **$400 - Answer: What is Benign Early Repolarization?**
  ○ ST elevations in V2–V6
  ○ No reciprocal depressions
- **$500 - Answer: What is acute pericarditis?**
  ○ Widespread concave ST elevation and PR depression is present throughout the precordial (V2–6) and limb leads (I, II, aVL, aVF)
  ○ Reciprocal ST depression and PR elevation in aVR only

**Indiana Pacemakers**

- **$100 - Answer: What is the right atrium?**
  ○ Will also accept: What are the atria?
  ○ Pacer spike precedes a P wave
  ○ This shows that the pacemaker is pacing the atria
  ○ QRS complex should be narrow as the impulse travels through the His-Purkinje system (except if there is a pre-existing LBBB or RBBB)
- **$200 - Answer: What is the right ventricle?**
  ○ Will also accept: What is the ventricle?
  ○ Pacer spike precedes the QRS complex
  ○ QRS is wide because pacer impulse enters myocardium outside the His-Purkinje system
- **$300 - Answer: What is failure to capture?**
  ○ Note the intermittent QRS complexes after each pacer spike
- **$400 - Answer: What is pacemaker induced tachycardia?**
  ○ Will also accept: What is runaway pacemaker?
  ○ The pacemaker is going about 250 bpm
  ○ What is a quick and easy way to treat this?
    ■ Answer: Apply a magnet → pacemaker goes into magnet mode, or a fixed paced rate usually at ~80 bpm
- **$500 - Answer: What is failure to sense?**
  ○ Notice the atrial beat with an inappropriately absent QRS
  ○ This is because the pacemaker failed to sense the absence of a QRS complex, and did not deliver an impulse

**Drugs and Bugs**

- **$100 - Answer: What are beta blockers?**
  ○ Will also accept: What are calcium channel blockers?
  ○ Sinus bradycardia
  ○ First degree AV block (prolonged PR interval)
- **$200 - Answer: What are tricyclic antidepressants?**
  ○ Wide QRS complex
  ○ Broad “terminal” R’ wave in aVR
- **$300 - Answer: What is Coxsackie B?**
  ○ Will also accept: What is Adenovirus? Or: What are Enteroviruses?
  ○ Viral myocarditis
  ○ Diffuse ST elevations with a convex upward (sad face) morphology
- **$400 - Answer: What is** ***Borrelia burgdorferi*** **?**
  ○ Erythema chronica migrans of Lyme disease
  ○ ECG shows 3^rd^ degree heart block, which can be seen in patients with early disseminated infection
- **$500 - Answer: What is digoxin?**
  ○ Frequent PVCs (most common ECG finding of dig toxicity)
  ○ Atrial tachycardia
  ○ 2nd degree Mobitz II block

## Appendix I Jeo-CARD-y Part 2 ECG Bootcamp Instructor Notes

**Notes to the Educator (PLEASE READ):**

*****This slideshow was created in PowerPoint and works best with PowerPoint features. It is fully viewable in Google Slides, but some animations, transitions, or formatting may not display exactly as intended.** Please download it to PowerPoint before opening it in Google slides.*******

1. Only half of the board is shown. This gives you 35 minutes to go through 15 prompts and allows time for teaching after each one. There is another station where the other 3 categories of Jeo-card-y are played.
2. Before you start, please divide each group into 3 separate smaller groups.
  - The reason for this is to encourage collaboration between group members.
  - Please make sure that each group has an even distribution of medical students, EM PGY-1s, 2s, 3s, and TY/Anesthesia PGY-1s.
3. Each of these 3 smaller groups will be a “contestant.” To make this more fun, each “contestant” can choose their own joke name and write it on a sheet of paper.
4. Randomly select which “contestant” goes first. They will choose a square on the board.
5. You, the educator, must be allowed to read the prompt in its entirety **BEFORE** a contestant is allowed to answer. If a contestant attempts to answer before you finish the prompt, they will be ignored.
6. For a contestant to indicate they are ready to answer the prompt, they must tap the desk in front of them. Whichever contestant you recognize first, is allowed to answer.
7. The answer to the prompt **must be in the form of a question**. Those answers that are not in the form of a question will be deemed incorrect.
8. Each correct answer adds the $ amount associated with the prompt to the contestant’s score. Each incorrect answer subtracts the $ amount associated with the prompt from the contestant’s score.
9. After each prompt is finished, please click “ **click to return to Jeo-CARD-y board**“ or the home button at the bottom to return to the game board. If you click anywhere else on the screen, it will advance you to the next prompt without allowing the contestant to choose which they want.
10. At the end of the game, the scores should be calculated as shown:
  **Table:**
  Raw Score	Points (out of 30)
  0–500	10
  600–1000	15
  1100–1500	20
  1600–2000	25
  2000+	30
11. At the end of each session, the mini team with the highest score will receive candy, and their score will be recorded for their larger team (4–6 players). These scores should be recorded and eventually summed across all stations to determine the final winning team for ECG Bootcamp.

**Answer Key:**

**Shock, Block, or Neither**

**To get credit, the learner must:**

1. **Correctly determine if the patient needs to be shocked, blocked (medicated), or neither**
2. **Correctly identify the rhythm in question format (i.e., What is normal sinus rhythm?)**

- **$100 - Answer: Shock! What is Vtach?**
  ○ Please note the BP
  ○ This is an example of monomorphic VT
  ○ V2 lead may be misplaced or not properly attached to the patient’s body
  ○ If you have VT with a pulse, then perform synchronized cardioversion 120 – 100J. If you have pulseless VT, then defibrillate at 120 – 100 J.
- **$200 - Answer: Shock! What is atrial flutter?**
  ○ There is a 4:1 block
  ○ Atrial rate of 260 bpm
  ○ Cardioversion is preferred over rate/rhythm controlling meds in new onset Aflutter
  ○ Rate/rhythm controlling meds can reduce efficacy of cardioversion!
- **$300 - Answer: Neither, treat the underlying cause! What is AF with RVR?**
  ○ Will also accept: What is treat the infection? What is treat the fever?
  ○ This ECG shows AF (atrial fibrillation) with RVR (rapid ventricular rate)
  ○ Patients with AF having RVR due to a fever is akin to a patient with sinus rhythm having sinus tachycardia due to a fever
    ■ Unless you feel that the HR is too fast to allow ventricular filling (and thus the reason for hypotension), try to avoid beta blockers or CCB
- **$400 - Answer: Either shock or block are acceptable. What is SVT with aberrancy?**
  ○ Will also accept: What is SVT with RBBB?
  ○ Wide complex tachycardia (WCT) in children and young adults is more than likely SVT with aberrancy, whereas WCT in older adults with multiple co-morbidities is more likely VT.
    ■ However, the general rule of thumb: If you’re in doubt about a widecomplex rhythm, treat it like VT (amiodarone +/− shock). Other clues to help you determine SVT with aberrancy: prior ECG showing a LBBB, RBBB, or WPW
- **$500 - Answer: Neither, treat the underlying cause. What is multifocal atrial tachycardia?**
  ○ 3 or more ectopic foci within the atria causing an irregular atrial tachycardia
    ■ P waves present; however, there are 3+ P wave morphologies
    ■ Irregular, and often mistaken for AF
  ○ Due to increased automaticity due to numerous causes, so it is important to treat that underlying cause
    ■ Infection/sepsis
    ■ COPD/CHF exacerbation
    ■ Electrolyte derangements (hypomag, hypokalemia)
    ■ PE
    ■ Anemia
    ■ DKA
    ■ Digoxin toxicity
  ○ Often refractory to cardioversion and AV nodal blocking agents

**The Real Deal (True STEMIs)**

- **$100 - Answer: What is an aVR elevation MI?**
  ○ Will also accept: What is a left main coronary artery (LMCA) occlusion?
  ○ ST-Elevation in aVR of 3 mm and diffuse reciprocal ST-Depressions (with exception of aVL and V1)
  ○ Recall: ST elevation (STE) must be > 1 mm with reciprocal depressions, AND have a history indicating ACS to meet criteria
- **$200 - Answer: What are positive Sgarbossa’s criteria?**
  ○ Modified Sgarbossa’s is applied to LBBB or a Ventricular paced rhythm (this is V paced):
    ■ Concordant STE ≥ 1 mm in ≥ 1 lead
    ■ Concordant ST depression ≥ 1 mm in ≥ 1 lead of V1–V3
    ■ Excessive discordant STE in ≥ 1 lead anywhere with ≥ 1 mm STE, as defined by ≥ 25% of the depth of the preceding S-wave
- **$300 - Answer: What are DeWinter’s T waves?**
  ○ Upsloping ST-Depression at J Point in leads V1 – V4 without STE
  ○ Tall, Symmetric T-Wave in leads V1 – V4
  ○ STE in lead aVR +/− aVL
- **$400 - Answer: What is a posterior MI?**
  ○ ***NOTE the posterior leads image on answer slide***
  ○ ST depression in V2–3
  ○ Tall, broad R waves (> 30 ms) in V2–3
  ○ Dominant R wave (R/S ratio > 1) in V2
  ○ Upright terminal portions of the T waves in V2–3
- **$500 - Answer: What is Wellen’s sign?**
  ○ Thought to be a STEMI that re-perfused, and what you are seeing is the electrical changes of that reperfusion
  ○ Type 1 (25%) biphasic T waves in V1–3 with slight ST elevations
  ○ Type 2 (75%) deeply inverted and symmetrical T waves in V1–V6 (see page 4 for an example of Type 2 Wellens)

**Nature vs. Nurture (Environmental and Peds)**

- **$100 - Answer: What are Juvenile T wave inversions?**
  ○ Commonly seen normal variant
  ○ No treatment needed
- **$200 - Answer: What is a RBBB-like morphology?**
  ○ Will also accept: What is a RBBB?
  ○ This does NOT represent a true RBBB. The QRS duration is normal, but it does show RSR’ not uncommon in this age group
  ○ Normal variant in babies and children
- **$300 - Answer: What is bi-directional Vtach?**
  ○ Will also accept: What is digoxin toxicity?
  ○ Bi-directional Vtach is pathognomonic for Dig toxicity
  ○ Note the alternating positive then negative QRS complexes
- **$400 - Answer: What are Osborne J Waves?**
  ○ Sometimes seen on ECGs of patients who are hypothermic
  ○ This is NOT pathognomonic, and can be seen in other things like:
    ■ Acute MI
    ■ Takotsubo cardiomyopathy
    ■ Hypercalcemia
    ■ LVH
    ■ Neurological insults (head injury, increased ICP)
    ■ Or just a normal variant (as seen in the next question!)
- **$500 - Answer: What is J-point notching?**
  ○ Can look similar to Osborne J waves
  ○ Normal variant seen in adolescents and young adults
  ○ No treatment is needed

## Appendix M Who Wants to be a Milliamp? ECG Bootcamp Instructor Notes

**Notes to the Educator (PLEASE READ):**

1. Upon opening the PowerPoint, click “Enable Macros.” If you accidentally clicked “Disable Macros,” please exit out and open it back up and click “Enable Macros.”
2. If a “Run Macros” message appears while using the PowerPoint, please click “Run Macros.”
3. For slide two titled, “How to Play,” before clicking “Play Game,” please allow a few seconds for all 3 lifelines to appear. They will not appear right away.
4. For each question, you will click an answer, which will be followed by “Is this your final answer,” for which you choose “Yes” or “No.” Correct answers will advance the learners; a wrong answer will end the game.
5. In the event that the learners answer a question incorrectly and the game ends, **please make note of where the game ended because this will determine their score**. Restart the game where they left off (helping them along the way if needed) until they go through all the questions. This will allow the learners to learn from all the material.
6. Visual instructions are provided by clicking the wrench in the bottom right corner of the PowerPoint’s title page.
7. You have 35 minutes to go through 15 questions and their respective teaching points. Please feel free to give any teaching pearls in addition to the bullet points in these notes.
8. Question 11 has 2 stimuli (see slideshow on Question 11 Follow Up Stimulus WWTBAM ECG Bootcamp):
  - Posterior MI seen via a conventional 12 lead
  - That same posterior MI seen via posterior leads
    - Please show the second ECG AFTER the learners answer the question.
9. All members of the group will be working together to allow for the most collaboration.
10. For the lifeline “phone a friend,” 1 participant may call anyone they want in the time allotted. Once that time has expired, the call must end. No more than 1 phone call is allowed.
11. Once the group has finished, scoring is calculated:
  **Table:**
  # Correct Answers (out of 15)	Points (out of 30)
  0–5	10
  6–8	15
  9–11	20
  12–13	25
  14–15	30
12. Please record that score so that a final winner for ECG boot camp can be determined!

**Answer Key:**

Question 1 - $100

32-year-old male presents with vomiting and palpitations. His ECG shows the following. All of the following medications are contraindicated except:

- Lorazepam
- Diphenhydramine
- Droperidol
- Ondansetron
  - This ECG shows hypokalemia with prolonged QT, U waves, and transient torsade de pointes

Question 2 - $200

In the ECG that is shown, which of the following findings is present (which is diagnostic of that type of AV block)?

- Consistent PR interval prolongation
- Progressively lengthening PR interval
- Random unpredictable dropped ventricular beat
- Complete atrioventricular dissociation
  - This ECG is an example of 2nd degree Mobitz I AV block

Question 3 - $300

All of the following are found in normal ECGs during childhood and/or adolescence except:

- T wave inversions in V1–V3
- Sinus arrhythmia
- LVH criteria
- RVH criteria
  - All other things listed in B-D are seen in normal pediatric ECGs

Question 4 - $500

Which of the following conditions does not result in a wide complex tachydysrhythmia?

- Wolff Parkinson White
- Right bundle branch block
- Brugada syndrome
- None of these
  - WPW can result in antidromic AVNRT (wide complex)
  - RBBB by definition has a wide QRS complex
  - Brugada can put patient at risk for spontaneous VT

Question 5 - $1,000

62-year-old female with HTN, DM, ESRD on HD, MI presents with fatigue for 2 days. She denies any chest pain, shortness of breath, diaphoresis, or nausea. Her ECG is shown. Which of the following is the next best step?

- Active a cardiac alert
- Administer oral aspirin
- Administer IV calcium gluconate
- Administer IV sodium bicarbonate
  - This ECG illustrates peaked T waves of hyperkalemia

Question 6 - $2,000

28-year-old female who is post-op day 5 s/p C-section due to failure to progress presents with sudden onset shortness of breath. Vital signs show tachycardia but are otherwise normal. ECG is shown. Which of the following is next best step?

- Repeat ECG in 15 minutes
- Obtain a CTA chest
- Obtain a troponin level
- Obtain a D-dimer
  - Possible pulmonary embolism in a postpartum patient
  - Most appropriate next step is CTA chest, not D-dimer because she has several risk factors for pulmonary embolism

Question 7 - $4,000

An 8-year-old male presents with palpitations and dizziness. He denies any other complaints. Vitals show tachycardia to 220 but are otherwise normal. ECG is shown. Which of the following the next best step?

- Vagal maneuvers
- IV Adenosine
- IV Esmolol
- Synchronized cardioversion
  - SVT in a child

Question 8 - $8,000

82-year-old female with a pacemaker 2 months ago presents with dizziness and syncope. Which of the following problems is her pacemaker most likely to have?

- The battery is low
- A pacemaker lead is intermittently displacing from the ventricular wall
- The sensing function of the pacemaker is too high
- The sensing function of the pacemaker is too low
  - This ECG strip illustrates failure to capture
  - The 3^rd^ pacer spike does not result ventricular depolarization
  - Almost any electrolyte derangement can cause failure to capture, but hyponatremia is not one of them

Question 9 - $16,000

This ECG finding may be seen in which of the following?

- Familial sodium channelopathy
- Familial potassium channelopathy
- C. Therapeutic digoxin use
- Hypothermia
  - Salvador Dali sign or Hockey stick sign
  - This is a normal variant in patients taking digoxin, and who have normal digoxin levels

Question 10 - $25,000

This ECG finding is most indicative of a problem of the:

- SA node
- AV node
- Left and right bundle branches
  - His-Purkinje system
  - Second degree Mobitz type II block
  - Recall this block is actually a misnomer in that the lesion is typically *below* the AV node

Question 11 - $50,000

While on shift, you are handed this ECG of a 62-year-old male with chest pain. Which of the following is the next best step?

- Activate a cardiac alert
- Order an aspirin and mark him for the next available bed
- Order posterior leads
- Indicate “no cardiac alert” and continue your work

Notable characteristics of a posterior MI include:

- ST depression in V2–3
- Tall, broad R waves (> 30 ms) in V2–3
- Dominant R wave (R/S ratio > 1) in V2
- Upright terminal portions of the T waves in V2–3

Follow up ECG showing posterior leads

- Posterior infarction is diagnosed based on the presence of ST segment elevation **>0.5 mm in leads V7–9**.
- Note that there is also some inferior STE in leads III and aVF (but no Q wave formation) suggesting early inferior involvement

Question 12 - $100,000

27-year-old female with depression is brought in by ambulance after an intentional overdose. Which of the following antidotes would most likely be indicated?

- Glucagon
- IV Sodium bicarbonate
- IV Magnesium sulfate
- Digibind
  - IV sodium bicarbonate is indicated to overcome the fast sodium channel blockade that leads to QRS widening and ventricular arrhythmias

Question 13 - $250,000

In the 3-letter nomenclature denoting pacemaker functionality, which of the following does the 3^rd^ letter indicate?

- Programmability
- Response to sensing
- The chamber that is paced
- The chamber that is sensed
  - First letter = chamber paced
  - Second letter = chamber sensed
  - Third letter = device’s response to sensing

Question 14 - $500,000

3-year-old male is brought in by his mother due to concern that he accidentally took some of his grandmother’s verapamil. ECG is as shown. All of the following are potential therapies except?

- Dobutamine
- Glucagon
- High dose insulin/glucose
- Atropine
  - Dobutamine is not a specific therapy for calcium channel blocker toxicity, and therefore does not address the bradycardia, contractility, and metabolic derangements caused by overdose

Question 15 - $1,000,000

The familial cardiomyopathy which this ECG finding indicates can best be described pathophysiologically as:

- A sodium channelopathy
- A potassium channelopathy
- Right ventricular myocardium replaced by non-conducting fibrofatty tissue
- Overgrowth of the ventricular septal myocardium
  - This ECG demonstrates the epsilon wave of arrhythmogenic right ventricular dysplasia Extra question if time permits:

All of the following criteria distinguish an ECG as Vtach as opposed to SVT with aberrancy except:

- Previous ECG showing delta waves
- Extreme Northwest Axis
- Atrioventricular dissociation
- Wide QRS complex
  - Wide QRS is a feature of both rhythms, whereas AV dissociation, extreme axis, and delta waves are specific findings used to differentiate VT from SVT with aberrancy

## Appendix N Code Blue Names Playing Cards

**Table:**

Imipramine	Cocaine
V Tach	Aspirin
Digoxin	T Wave Inversions
Narrow Complex	J Point Elevation
Cardioversion	Adenosine
Q Waves	Congenital Long QT
Magnesium	Sodium Bicarbonate
Overdrive Pacing	PVCs
Peaked T Waves	AV Block
Brugada	Torsades De Pointes
Sgarrbossa	Wellen’s Sign
Biphasic T Waves	Sinus Rhythm
Hypertrophic Cardiomyopathy	Blue Team
Blue Team	Blue Team
Blue Team	Blue Team
Blue Team	Blue Team
Blue Team	Blue Team
Red Team	Red Team
Red Team	Red Team
Red Team	Red Team
Red Team	Red Team
Neutral	Neutral
Neutral	Neutral
Neutral	Neutral
Neutral	
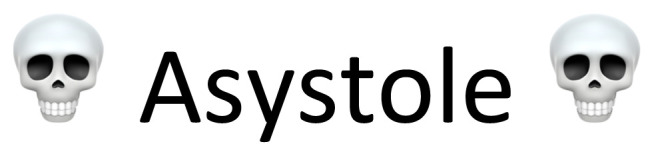

## Appendix P Code Blue Names Instructor Notes

General notes to the Educator:

1. The object of the game is to be the first team to correctly identify your team’s assigned cards on a 50×5 grid of terms.
2. There will be two sub-teams (Red and Blue) where members of each sub-team will be assigned to one of two roles: Clue Giver or Guesser.
3. Clue Givers on each team will look at the Code Blue Names Color Card, identify which cards on the 5×5 grid belong to their team, and guide their team’s Guessers to correctly identify these cards by providing clues.
  - The clue givers can give a clue that is up to 2 words in length AND a number that corresponds to the number of cards they are indicating for that clue.
    - Example: Clue Giver says “Channelopathy, 2.” Guessers correctly choose the cards “Congenital Long QT” and “Brugada.”
    - Be careful that clues given don’t apply to the opposing team’s cards, neutral cards, or the asystole card.
    - Ensure that clues given are fair and attempt to associate the content of the cards together (no clues referring to the card’s position on the grid, rhyming, etc. )
4. The winning sub-team (Red or Blue) should be awarded candy or a small treat but note that the points calculated at the end for the winning sub-team will be awarded to the team as a whole!
  - As described in the instructions section below, winning in fewer turns means more points. This will incentivize critical thinking to attempt to associate more of their team’s cards together in one turn.
5. This video may be a helpful resource to learn the basic mechanics of the game. Note that here, Spymaster = Clue Giver, Bystander = Neutral, and Assassin = Asystole. Also, our modified version of the game includes multiple Clue Givers instead of just one Spymaster per team like the video says.
6. Educational Goals:
  - Reinforce recognition of key ECG patterns and cardiac conditions
  - Improve clinical reasoning by connecting ECG findings to diagnoses and treatments
7. Timing:
  - Each team should spend no more than 2–3 minutes per turn (including discussion and guessing). This pacing ensures the game can be completed within the allotted 35-minute session.

Game Preparation:

1. Print out Code Blue Names Playing Cards and cut out all 50 cards individually.
2. Lay out the first 25 terms in a 5×5 grid exactly as it appears in the ECG Code Blue Names Master Template. **This is very important because it directly corresponds to the** **Code Blue Names Color Card** **which the Clue Givers will use to guide their teams to the correct cards.** The purpose of the other 25 cards (Red Team, Blue Team, Neutral, and Asystole) will be explained in the game instructions below.
3. Print out Code Blue Names Color Card (**This is for Clue Givers’ eyes only!**)

Game Instructions:

1. Break the group up into the Red Team and Blue Team. Half of the members of each team will serve as Clue Givers, and the other half will serve as Guessers. Clue Givers may collaborate together to decide on the clue given to their team each round.
2. Clue Givers on each team will look at the Code Blue Names Color Card and identify which cards correspond to their team. Out of 25 cards, there are 9 blue cards, 8 red cards, 7 neutral cards, and 1 asystole card.
  - **Only the Clue Givers should be allowed to view the Code Blue Names Color Card**. If the Guessers see it, that constitutes cheating and defeats the purpose of the game.
3. The Blue Team’s Clue Givers will start by giving their Guessers a clue containing up to 2 words and a number that corresponds to how many cards that clue applies to.
  - Guessers will then work together to collectively choose cards by touching the card on the grid.
  - Guessers may make up to one more guess than the number given in the clue (e.g., a clue of “Channelopathy, 2” allows up to 3 guesses).
  - Guessers are not required to use all guesses associated with the clue and can stop at any time if they are unsure.
4. The card’s “identity” will then be revealed as Red Team, Blue Team, Neutral, or Asystole and marked accordingly by laying the designated label card on top of the term (found in Code Blue Names Playing Cards)
  - If a Guesser correctly chooses a card that belongs to their team, they may continue guessing until they either reach the allowed guess limit, select an incorrect card, or decide to stop.
  - If a Guesser incorrectly chooses a card that belongs to the opposing team or a neutral card, that team’s turn is over.
  - If a Guesser incorrectly chooses the deadly “asystole card” (represented by VTach in this case), **that team automatically loses the game.**
5. Teams will take turns in providing clues and making guesses until one team correctly identifies all of their team’s cards or until the opposing team mistakenly selects the asystole card which ends the game.
6. Award the winning team candy or a small treat, then compile the points for the entire team based on the winning team’s performance as follows:
  - Scoring (Maximum = 30 points):
    - Winning team (Red or Blue) receives **20 base points**
    - Additional points are awarded based on number of turns required to win:
      1. 1–4 turns: +10
      2. 5–6 turns: +7
      3. 7–8 turns: +5
      4. 9+ turns: +3
      5. If the game ends due to the opposing team’s selecting the Asystole card, subtract 5 points from the total score.

Further Reading:

1. How to Play Codenames. Youtube. March 30, 2021. Accessed March 16, 2026. https://www.youtube.com/watch?v=WErB95Brgbs.

## Appendix Q Code Blue Names Master Template

**Table:** ECG Code Blue Names Master Template

Imipramine	PVCs	Digoxin	Wellen’s	Sign Adenosine
Sgarbossa	Sodium Bicarbonate	Brugada	Peaked T waves	Magnesium
J point elevation	AV block	Q waves	Narrow complex	Aspirin
Cardioversion	V Tach	Overdrive pacing	Sinus arrythmia	Torsades de pointes
Hypertrophic cardiomyopathy	Congenital Long QT	T wave inversions	Biphasic T waves	Cocaine

Brown is Neutral

Black is Asystole

Red is Red Team

Blue is Blue Team

## Appendix R Torsades Simulation Case

**Case Title:** Torsade de pointes with ECG Evaluation

**Time required for implementation:**

**Preparation:** 20 minutes
**Time for case:** 5 minutes for prebrief, 20 minutes for case
**Time for debriefing:** 10 minutes

**Recommended number of learners per instructor/case:** Number of residents on a team should be anywhere from 5–10.

**Recommended pre-reading for instructor:** Recommend having familiarity with the guidelines for treating torsades de pointes and reviewing the best practices for high-fidelity simulation. A recommended reading: Cohagan B, Brandis D. Torsade de pointes. *Stat Pearls.* Updated 8 August 2023. https://www.ncbi.nlm.nih.gov/books/NBK459388/

**Objectives:**

By the end of this case, learners will be able to:

1. Correctly identify QT prolongation on ECG and initiate cardiac monitoring.
2. Recognize polymorphic ventricular tachycardia consistent with torsade de pointes and treat with both first-line and second-line interventions.
3. Accurately triage rapidly presented ECGs within 20 seconds as requiring a cardiac alert, monitoring required, or no intervention.

**Tips for successful implementation:** You may need to print several copies of the ECGs (or laminate them) to ensure they endure the total day when running this multiple times.

**Case Title:** Torsade de pointes with ECG Evaluation

**Case description & Diagnosis (short synopsis):** A 32-year-old man with daily cannabis use presents with 12 hours of intractable nausea and vomiting and recent emergency department discharge on ondansetron. He is tachycardic and appears dehydrated, with ongoing emesis and mild epigastric tenderness. Early evaluation reveals a prolonged QT interval on ECG, and despite initial management, he develops intermittent episodes of polymorphic ventricular tachycardia with hemodynamic instability. Laboratory studies later demonstrate significant electrolyte abnormalities, including hypokalemia and hypomagnesemia, in the setting of persistent vomiting. The patient is diagnosed with torsades de pointes secondary to acquired QT prolongation, precipitated by QT-prolonging medication use (ondansetron) and electrolyte derangements. Participants will have to treat with magnesium sulfate and then isoproterenol when magnesium alone proves to be ineffective in order to prevent the patient from going into cardiac arrest. Throughout the case, an ER technician will be presenting the participants with 10 ECGs of other ER patients that participants will have to appropriately triage in order to prevent further risk to the department.

**Equipment or Props Needed:**

high-fidelity manikin, defibrillator pads and cardiac monitor, IV pole, simulated medications of IV fluids, antiemetics, magnesium sulfate, isoproterenol, and ACLS cart if necessary.

**Actors needed**

- ED technician: Needed to present participants with 10 separate ECGs
- ED nurse

**Stimulus Inventory: (See Appendix S)**

**Table:**

#1	CXR
#2	KUB
#3	Initial ECG – sinus tachycardia with prolonged QT
#4	Second ECG - Prolonged QT that degenerates into torsade de pointes
#5	Third ECG – ECG after isoproterenol
#6	CBC
#7	CMP + Mag
#8	Coags
#9	Lactate
#10	Troponin

**Background and brief information:** 32-year-old male with a history of daily cannabis use presents to a tertiary care facility with intractable nausea and vomiting.

**Initial presentation:** Patient is ill-appearing, dehydrated, and retching throughout exam.

**How the scenario unfolds:** The patient will be placed on a cardiac monitor, and an initial ECG will be obtained revealing a prolonged QT interval. Soon after, the patient will develop intermittent runs of polymorphic ventricular tachycardia concerning for torsade de pointes. Participants will have to give the patient magnesium sulfate, and when that is unsuccessful in treating the torsades, administer isoproterenol in order to prevent the patient from decompensating into cardiac arrest. They will be unable to consult cardiology or pharmacy throughout the scenario. If attempts are made to place a transvenous pacer for overdrive pacing, the patient will further decompensate. Throughout the case, an ER technician will be coming in and showing the participants the ECGs of 10 other patients in the emergency department, forcing the participants to rapidly triage other patients to prevent further risk.

**Critical actions:**

1. Recognize QT prolongation on initial ECG – place the patient on a cardiac monitor.
2. Avoid administration of QT-prolonging medications (i.e., ondansetron, haloperidol).
3. Administer slow 2 gram IV push of magnesium sulfate promptly.
4. Escalate care for unstable arrhythmia – apply defibrillator pads, prepare for ACLS interventions, perform synchronized cardioversion.
5. Initiate second-line therapy with isoproterenol and correct underlying causes of hypokalemia and hypomagnesemia.

**Case Title:** Torsade de pointes with ECG Evaluation

**Chief Complaint:** Nausea and vomiting

**Table:**

**Vitals:**	Heart rate (HR)112	Blood pressure (BP) 104/76	Respiratory rate (RR)12
	Temperature (T) 36.8° C	Oxygen saturation (O_2_Sat) 99% Room Air (RA)	

**General Appearance:** Ill-appearing, in moderate distress, actively vomiting

**Primary Survey:**

- **Airway:** Open
- **Breathing:** Bilateral breath sounds
- **Circulation:** Tachycardic, decreased peripheral pulses, strong central pulses

**History:**

- **History of present illness:** 32-year-old male with a history of daily cannabis use presents with intractable nausea and vomiting. He denies vomiting any bile or blood. No associated abdominal pain. He states that he has been unable to hold anything down for the past 12 hours without relief. He denies any fever, chest pain, cough, diarrhea, bloody stool, rash, sick contacts or recent travel. He recently visited the ER 3 days ago for a similar complaint, was prescribed ondansetron, and discharged home. He says the vomiting has not improved despite around the clock use of ondansetron.
- **Past medical history:** None
- **Past surgical history:** None
- **Patient’s medications:** Ondansetron
- **Allergies:** No known drug allergies
- **Social history:** Daily marijuana use, social alcohol use, no tobacco use
- **Family history:** No pertinent family history

**Secondary survey/physical examination:**

- **General appearance:** Retching, in moderate distress
- **HEENT:**
  ○ **Head:** Within normal limits (wnl)
  ○ **Eyes:** wnl
  ○ **Ears:** wnl
  ○ **Nose:** wnl
  ○ **Throat:** wnl
- **Neck:** wnl
- **Heart:** Tachycardic, otherwise wnl
- **Lungs:** wnl
- **Abdominal/GI:** No significant abdominal tenderness to palpation, no rebound or guarding, normoactive bowel sounds
- **Genitourinary:** wnl
- **Rectal:** wnl
- **Extremities:** Decreased capillary refill, otherwise, wnl
- **Back:** wnl
- **Neuro:** wnl
- **Skin:** decreased skin turgor, otherwise wnl
- **Lymph:** wnl
- **Psych:** wnl

**Table:** SIMULATION EVENTS TABLE:

Minute (State)	Participant Action/Trigger	Patient Status (Simulator Response) & Operator Prompts	Monitor Display (Vital Signs)
0:00 (Baseline)	Participants walk in after being asked to evaluate an actively vomiting patient.	Patient actively retching, asking for something to help the nausea and vomiting.	T 36.8° C HR 112 BP 104/76 RR 12 O2 99% RA
2:00	Participants ask nurse to place patient on a cardiac monitor and start an IV.	Nurse asks for medications for the persistent emesis. Tech presents ECG #1 to the participants.	T 36.8° C HR 112 BP 104/76 RR 12 O2 99% RA
4:00	Participants order antiemetic for patient. Participants order IV fluids. Participants order acute abdominal series, ECG and labs.	If antiemetic is anything that prolongs the QT, the patient will decompensate into intermittent episodes of torsade de pointes. Tech presents ECG #2 to the participants.	T 36.8° C HR 112 BP 104/76 RR 12 O2 99% RA
6:00		ECG results (Stimulus #3). Tech presents ECG #3 to the participants.	T 36.8° C HR 112 BP 104/76 RR 12 O2 99% RA
8:00	Participants note long QT on ECG and order 1–2g IV magnesium.	Acute abdominal series (Stimuli #1 and #2) results. If participants do not give magnesium, nursing staff can ask questions such as, “Does the ECG show anything?” “Anything we can do about that?” Tech presents ECG #4 to the participants.	T 36.8° C HR 112 BP 104/76 RR 12 O2 99% RA
10:00		Patient starts to develop intermittent episodes of torsade de pointes regardless of antiemetic given. Nurse will state “Wow, it looks like there’s something weird going on the monitor; I’m going to get an ECG.” Tech presents ECG #5 to the participants.	T 36.8° C HR 112 (now with intermittent episodes of torsades) BP 104/76 RR 12 O2 99% RA
12:00	Participants ask about magnesium, which has been given.	ECG available (Stimulus #4) Patient starts to complain more, and becomes more altered and lethargic, stating, “I don’t feel good.” Tech presents ECG #6 to the participants.	T 36.8° C HR 112 (now with intermittent episodes of torsades) BP 80/50 RR 28 O2 90% RA
14:00	Oxygen started. Synchronized cardioversion performed. IV fluids started.	Labs available (Stimuli #6–10) Tech presents ECG #7 to the participants.	T 36.8° C HR 112 (now with intermittent episodes of torsades) BP 104/76 RR 22 O2 94% RA
16:00	Magnesium infusion started. Recognize hypokalemia and administer potassium supplementation.	Nurse states “It looks like he’s still in torsades – anything else we can do?” Tech presents ECG #8 to the participants.	T 36.8° C HR 112 (now with intermittent episodes of torsades) BP 104/76 RR 22 O2 94% RA
18:00	Isoproterenol given.	Repeat ECG available (Stimulus #5) Tech presents ECG #9 to the participants.	T 36.8° C HR 112, sinus tach BP 104/76 RR 12 O2 94% RA
20:00	Case ends.	Tech presents ECG #10 to the participants. Patient is admitted to the ICU for further management, may allow presentation to the ICU if time permits.	Case ends.

**Diagnosis:** Torsade de pointes, likely medication-induced

**Disposition:** ICU

### DEBRIEFING AND EVALUATION: Torsade de Pointes^11^

**What it is:** form of polymorphic ventricular tachycardia in which the QRS complexes on ECG vary in size and appear to twist around the baseline

**Caused by:** QTc prolongation

**ED Management Pearls:**

Identify and remove the trigger early

- Discontinue all QT-prolonging medications and address reversible causes (e.g., vomiting, drug effects).

Correct electrolytes aggressively

- Replete **K****^+^**** to 4.5–5.0 mmol/L** and normalize magnesium and calcium levels to reduce recurrence risk.

Magnesium is first-line - regardless of serum level

- Give **2 g IV magnesium sulfate** (slow push), followed by infusion if needed; stabilizes cardiac membrane even if Mg is normal.

Treat instability like any other malignant arrhythmia

- **Unstable with pulse → synchronized cardioversion**
- **Pulseless → defibrillation + ACLS**

Increase heart rate for refractory torsades

- Use **isoproterenol (less invasive) or overdrive pacing (more invasive)** to shorten QT and suppress arrhythmia recurrence.

Do not delay treatment for labs

- Torsades is a **clinical/ECG diagnosis**—initiate magnesium and resuscitation immediately.

Anticipate recurrence even if self-terminating

- Episodes may stop spontaneously but can **degenerate into ventricular fibrillation**, so continuous monitoring is essential.

**ECG Interpretation in the Emergency Department**

Ask yourself 3 questions:

1. Is this a STEMI? If so – activate a cardiac alert.
2. Are there any ischemic changes that do not meet STEMI criteria? If so – place the patient on a cardiac monitor.
3. Is there an arrhythmia? If so – place the patient on a cardiac monitor.

If none of the above are true – patient does not need a cardiac monitor.

## Appendix U Answer Key for ECGs in Torsades Simulation Case

Patient information should be provided if asked.

**ECG #1:** 82 M with chest pressure. No prior EKG.

Correct response: Place on a cardiac monitor (due to ST segment depressions in the lateral leads)

**ECG #2:** 10F with palpitations.

Correct response: No cardiac alert. (J-point elevation – benign finding)

**ECG #3:** 45F with shortness of breath.

Correct response: Place on a cardiac monitor (due to S1Q3T3)

**ECG #4:** 64F with abdominal pain.

Correct response: No cardiac alert. RBBB on ECG.

**ECG #5:** 70M with chest pain.

Correct response: Cardiac alert! aVR elevation MI.

**ECG #6:** 62F with palpitations.

Correct response: Place on a cardiac monitor (due to atrial flutter)

**ECG #7:** 68M with nausea and vomiting.

Correct response: No cardiac alert. (LVH with strain)

**ECG #8:** 18M with palpitations.

Correct response: Place on a cardiac monitor (due to HOCM)

**ECG #9:** 56M with chest pain.

Correct response: Cardiac alert! Anterolateral STEMI.

**ECG #10:** 90M with chest pain.

Correct response: No cardiac alert.

## Appendix V Torsades Simulation Case Score Sheet

**Critical Action Items** (5 points per action completed)

_____ Recognize QT prolongation on initial ECG – place the patient on a cardiac monitor.
_____ Avoid administration of QT-prolonging medications (i.e., ondansetron, haloperidol).
_____ Administer slow 2 gram IV push of magnesium sulfate promptly.
_____ Escalate care for unstable arrhythmia – apply defibrillator pads, prepare for ACLS interventions, perform synchronized cardioversion.
_____ Initiate second-line therapy with isoproterenol and correct underlying causes of hypokalemia and hypomagnesemia.

**ECG interpretations** (2 points per correct ECG interpretation)

_____ ECG#1 (answer: cardiac monitor)
_____ ECG#2 (answer: no cardiac alert)
_____ ECG#3 (answer: cardiac monitor)
_____ ECG#4 (answer: no cardiac alert)
_____ ECG#5 (answer: cardiac alert)
_____ ECG#6 (answer: cardiac monitor)
_____ ECG#7 (answer: no cardiac alert)
_____ ECG#8 (answer: cardiac monitor)
_____ ECG#9 (answer: cardiac alert)
_____ ECG#10 (answer: no cardiac alert)

## Appendix W ECG Bootcamp Curriculum Chart

**Table:**

Topic	Recommended Educational Strategy	Appendices Needed	Timing (Prep and Activity)	Resources/Prep Needed (Space, Instructors, Equipment)
Introduction	Introductory PowerPoint to ECG Bootcamp. Includes learning objectives, agenda with station rotations, and brief descriptions of each game.	Appendix A: Introduction to ECG Bootcamp Appendix B: ECG Bootcamp Score Card	Preparation: None PowerPoint: 10 minutes	Divide learners into 6 teams. Print and cut out 6 ECG Bootcamp score cards and distribute 1 to each team. One instructor is needed.
ECG Association Game	Learners will “associate” ECGs together based on a clue provided by the instructor, containing a word/phrase and a number of ECGs to which the clue will apply.	Appendix C: ECG Association Game PowerPoint Appendix D: ECG Association Game Instructor Notes	Preparation: 15–20 minutes (read through instructor notes). Game: 2–3 minutes per round 35 minutes total	Table large enough to fit 16 8.5”x11” pieces of paper. Print the 16 ECGs (slides 2–17 from Appendix C: ECG Association Game PowerPoint), lay out in 4x4 grid on table. Print out 1 copy of the ECG Association Game Instructor Notes. One instructor is needed.
ECG Jeo-Card-y Part 1	ECGs are presented as clues, and participants must identify the correct pattern, diagnosis, or mimic. Categories include Indiana pacemaker, STEMI mimics, drug-induced changes, and infection-related findings (“drugs and bugs”).	Appendix E: Jeo-CARD-y Part 1 ECG Bootcamp PowerPoint Appendix F: Jeo-CARD-y Part 1 ECG Bootcamp Instructor Notes Appendix G: Jeo-CARD-y Part 1 Score Sheet ECG Bootcamp	Preparation: 15–20 minutes (read through instructor notes). Game: 35 minutes to go through 15 prompts (2–3 minutes per prompt). Allow time for teaching after each prompt.	Computer to access Microsoft PowerPoint application, with Jeo-CARDy Part 1 ECG Bootcamp PowerPoint on the computer. Table and chairs for instructor and participants. Print out 1 copy of the Jeo-CARD-y Part 1 ECG Bootcamp Instructor Notes. Print out 6 copies of the Jeo-CARD-y Part 1 Score Sheet ECG Bootcamp. ***This slideshow was created in PowerPoint and works best with PowerPoint features. It is viewable in Google Slides, but some animations, transitions, or formatting may not display exactly as intended.*** One instructor is needed. Candy/treat for the winning sub-team.
ECG Jeo-CARD-y Part 2	ECGs are presented as clues paired with brief clinical scenarios, and participants must identify the correct rhythm, diagnosis, or pathology. Categories include Shock, Block, or Neither, The Real Deal (True STEMIs, and Nature vs Nurture, distinguishing normal variants from pathology).	Appendix H: Jeo-CARD-y Part 2 ECG Bootcamp PowerPoint Appendix I: Jeo-CARD-y Part 2 ECG Bootcamp Instructor Notes Appendix J: Jeo-CARD-y Part 2 Score Sheet ECG Bootcamp	Preparation: 15–20 minutes (read through instructor notes). Game: 35 minutes to go through 15 prompts (2–3 minutes per prompt). Allow time for teaching after each prompt.	Computer to access Microsoft PowerPoint application. Table and chairs for instructor and participants. Print out 1 copy of the Jeo-CARD-y Part 2 ECG Bootcamp Instructor Notes. Print out 6 copies of the Jeo-CARD-y Part 2 Score Sheet ECG Bootcamp. ***This slideshow was created in PowerPoint and works best with PowerPoint features. It is viewable in Google Slides, but some animations, transitions, or formatting may not display exactly as intended. *** One instructor is needed. Candy/treat for the winning sub-team.
Who Wants to be a Milliamp?	This is an interactive quiz session where learners answer 15 progressively challenging ECG interpretation questions. Participants move from basic rhythm identification to complex arrhythmias and emergent findings, using limited “lifelines” to reinforce collaborative learning and clinical reasoning.	Appendix K: Who Wants to Be a Milliamp? ECG Bootcamp PowerPoint Appendix L: Question 11 Follow Up Stimulus for Who Wants to Be a Milliamp? ECG Bootcamp PowerPoint Appendix M: Who Wants to Be a Milliamp? ECG Bootcamp Instructor Notes	Preparation: 15–20 minutes (read through instructor notes). Game: 35 minutes to go through 15 prompts (2–3 minutes per prompt). Allow time for teaching after each prompt.	Computer to access Microsoft PowerPoint application. Print out 1 copy of Who Wants to Be a Milliamp? ECG Bootcamp Instructor Notes. Print out 1 copy of Stimuli for Who Wants to Be a Milliamp? ECG Bootcamp PowerPoint (or have this on a separate computer than the one showing the game show). Table and chairs for instructor and participants. ***The game slideshow was created in PowerPoint and works best with PowerPoint features. Please download it to the PowerPoint desktop application.*** One instructor is needed.
ECG Code Blue Names	Two sub-teams (Red vs. Blue) will compete to identify their assigned terms on a 5×5 grid by deciphering clues that attempt to connect them together. Topics include ECG abnormalities, arrhythmias, cardiac disease, ischemic patterns, cardiac drugs, toxicology, and treatments.	Appendix N: Code Blue Names Playing Cards Appendix O: Code Blue Names Color Card Appendix P: ECG Code Blue Names Instructor Notes Appendix Q: ECG Code Blue Names Master Template	Preparation: 15–20 minutes (read through Appendix P: Instructor Notes). Game: 2–3 minutes per round until one team wins, 35 minutes total	Print and cut out individual Code Blue Names Playing Cards (Appendix N), and lay out the cards with medical terms (i.e., not the cards with “Blue Team,” “Red Team,” “Neutral,” or “Asystole” - these will be for the participants when playing the game) in the order as described in Appendix Q: ECG Code Blue Names Master Template in a 5x5 grid on a table. Table large enough to contain the grid as described above, with chairs for each participant and instructor. Print out Appendix O: Code Blue Names Color Card, and give to the team members that will be providing the clues to their teammates. Print out Appendix P and Appendix Q for use by the instructor. Computer/phone/laptop/tablet to show video *** This video may be helpful to show beforehand to explain the basic mechanics of the game.*** Candy/treat for the winning sub-team. One instructor is needed.
Torsades Sim Case	This is a traditional simulation session where participants will have to manage a critically ill patient suffering from torsade de pointes while having to evaluate the ECGs of 10 other patients in the emergency department.	Appendix R: Torsade de Pointes Simulation Case Appendix S: Stimuli for Torsade de Pointes Simulation Case Appendix T: ECGs for Simulation Case Appendix U: ECG Answer Key Appendix V: Simulation Case Score Sheet	Preparation: 20 minutes Timeline: - Prebrief: 5 minutes - Sim Case: 20 minutes - Debrief: 10 minutes	Two instructors needed here - one to facilitate, and one to act as an ER tech bringing participants EKGs. Simulation technician needed. If available, an additional participant available to play the role of the ER nurse increases the fidelity of the case. High-fidelity manikin with monitor needed - remaining specific simulation needs detailed in the case itself. Print out 3 copies of Appendix R - one each for each instructor and the simulation technician. Either print out or upload Appendix S for display during the simulation case. Print out Appendix T for use during the simulation session, 1 slide on each page. Print out 1 copy of Appendix U for use by the ER technician. Print out 1 copy of Appendix V for each team that is participating in ECG Bootcamp.
Conclusion	Participants have the opportunity to finalize scoring and ask questions. The winning team is announced.	No supplies needed	Prep: Add up scores from all 6 games to choose winner. Time allotted: 10 minutes	One instructor is needed. Can have a prize for the winning team - may use the logo created for ECG Bootcamp if you’d like!
