# Supplementary material for: ECG Bootcamp - A Novel Emergency Medicine Small Group Session
Source: J Educ Teach Emerg Med. 2024 Jun 14;11(3):SG69–SG142. doi: 10.5070/M5.52155 (PMC13441231; doi:10.5070/M5.52155)
Supplement: Supplementary file 1 [file 11-3-SG69-Appendix_B.pdf]

ECG BOOTCAMP

WHO WANTS TO BE A MILLIAMP?....................................	POINTS:
ECG JEO-CARD-Y PART 1	POINTS:
ECG JEO-CARD-Y PART 2	POINTS:
SIM CASE	POINTS:
ECG CODE BLUE NAMES	POINTS:
ECG ASSOCIATION GAME	POINTS:
TOTAL POINTS...	POINTS:
