# Supplementary material for: ECG Bootcamp - A Novel Emergency Medicine Small Group Session
Source: J Educ Teach Emerg Med. 2024 Jun 14;11(3):SG69–SG142. doi: 10.5070/M5.52155 (PMC13441231; doi:10.5070/M5.52155)
Supplement: Supplementary file 2 [file 11-3-SG69-Appendix_N.docx]

| Imipramine | Cocaine |
| --- | --- |
| V Tach | Aspirin |
| Digoxin | T Wave Inversions |
| Narrow Complex | J Point Elevation |
| Cardioversion | Adenosine |

| Q Waves | Congenital Long QT |
| --- | --- |
| Magnesium | Sodium Bicarbonate |
| Overdrive Pacing | PVCs |
| Peaked T Waves | AV Block |
| Brugada | Torsades De Pointes |

| Sgarrbossa | Wellen’s Sign |
| --- | --- |
| Biphasic T Waves | Sinus Rhythm |
| Hypertrophic Cardiomyopathy | Blue Team |
| Blue Team | Blue Team |
| Blue Team | Blue Team |

| Blue Team | Blue Team |
| --- | --- |
| Blue Team | Blue Team |
| Red Team | Red Team |
| Red Team | Red Team |
| Red Team | Red Team |

| Red Team | Red Team |
| --- | --- |
| Neutral | Neutral |
| Neutral | Neutral |
| Neutral | Neutral |
| Neutral | 💀 Asystole 💀 |
